# Rectal Angiosarcoma: A Case Report Highlighting Multidisciplinary Strategies for Rare Malignancies

**DOI:** 10.3390/reports8020067

**Published:** 2025-05-15

**Authors:** Dan Corneliu Jinga, Sabina Sucuri, Irina M. Cazacu-Croitoru, Barhala Mihai, Bogdan Chivu

**Affiliations:** 1 Neolife Medical Center Bucharest, 077190 Bucharest, Romania; mihai.barhala@gmail.com; 2 Coltea Clinical Hospital Bucharest, 030167 Bucharest, Romania; 3 Fundeni Clinical Institute, 022328 Bucharest, Romania; irina.cazacu89@gmail.com

**Keywords:** rectal angiosarcoma, radiation therapy, rare cancers

## Abstract

**Background and Clinical Significance**: Rectal angiosarcoma is an exceptionally rare and aggressive malignancy, comprising less than 1% of soft tissue sarcomas. This case highlights the diagnostic and therapeutic challenges posed by this disease, and the lack of established guidelines emphasizing the importance of a multidisciplinary approach. **Case Presentation**: A 41-year-old male firefighter, with a history of heavy smoking, presented with lower abdominal pain, rectal bleeding, and urgency. Imaging and biopsy confirmed rectal angiosarcoma, stage IIIB. The patient underwent IMRT/VMAT radiation therapy followed by laparoscopic rectal amputation with colostomy. No sign of recurrence or metastatic disease was present on follow-up imaging. **Conclusions**: This case underlines the importance of a personalized treatment strategy and multidisciplinary collaboration in rare malignancies. Early diagnosis and cooperation across specialties are critical for achieving the best possible outcomes.

## 1. Introduction and Clinical Significance

Rectal angiosarcoma is a distinctly aggressive vascular tumor, accounting for less than 1% of all soft tissue sarcomas and 0.001% of all colorectal malignancies, making it a very rare finding [1]. Literature reviews report the first case of colorectal angiosarcoma in 1949, described by Steiner and Palmer [2], with approximately 40 cases being reported to date, most of them primary [3,4,5]; thus, due to its scarce presentation, there are no standardized guidelines for diagnosis and management, and its high aggressive course complicates the development of an optimal therapeutic approach. Etiologically, the causes of this rare malignancy remain unclear, yet it may be linked to prolonged exposure to radiation (previous radiotherapy treatment), exposure to polyvinylchloride, thorotrast and arsenic, along with the ongoing effects of chronic lymphedema and amyloidosis [6,7,8]. Furthermore, the association with Dacron vascular grafts, orthopedic joint prostheses, breast implants and arteriovenous fistulas was noted. Most of the colorectal angiosarcomas emerge in the sigmoid colon, with the rectum being the second most common site, which is the case of the patient presented herein (Wang et al. [3]). While starkly contrasting in terms of aggressiveness with the more common presentation of rectal adenocarcinomas, and as a consequence of its nonspecific presentation, overlapping symptomatology and infrequent occurrence of angiosarcomas in the gastrointestinal tract, this diagnosis is often delayed and requires a complex approach [1,9]. A 41-year-old man with rectal angiosarcoma is discussed further in this case report highlighting his diagnostic journey and treatment along with the importance of advanced imaging techniques, meticulous radiation planning, precise surgical intervention and the key role of the multidisciplinary tumor board review in guiding each therapeutic procedure.

The 5-year survival rate of angiosarcomas is close to 35%, accounting for the poor prognosis. Even with the most optimistic assessment in localized disease, only 60% of patients survive beyond 5 years, with a median lifespan of just 7 months [1,10]. However, some reports document cases of long-term survival in metastatic patients, though the prognostic markers still remain ambiguous [11]. Most available data regarding these rare malignancies derive from case series, as angiosarcomas are often included as a limited number of cases within comprehensive studies of all soft-tissue sarcomas [12].

## 2. Case Presentation

### 2.1. Clinical History

A 41-year-old male firefighter with a history of heavy smoking for over 20 years presented in 2023 with lower acute abdominal pain, constipation, rectal urgency and bleeding—symptoms ignored for more than 2 years.

In February 2023, a rectal examination revealed a firm tumor in the anal canal, measuring approximately 5–6 cm, with a high risk of bleeding upon contact and a free rectal ampulla. It is important to note that the patient had no history of prior prostate or pelvic radiation therapy, which is relevant given the known association between radiation exposure and the development of angiosarcomas [9].

### 2.2. Imaging and Diagnosis

**Figure 1:**
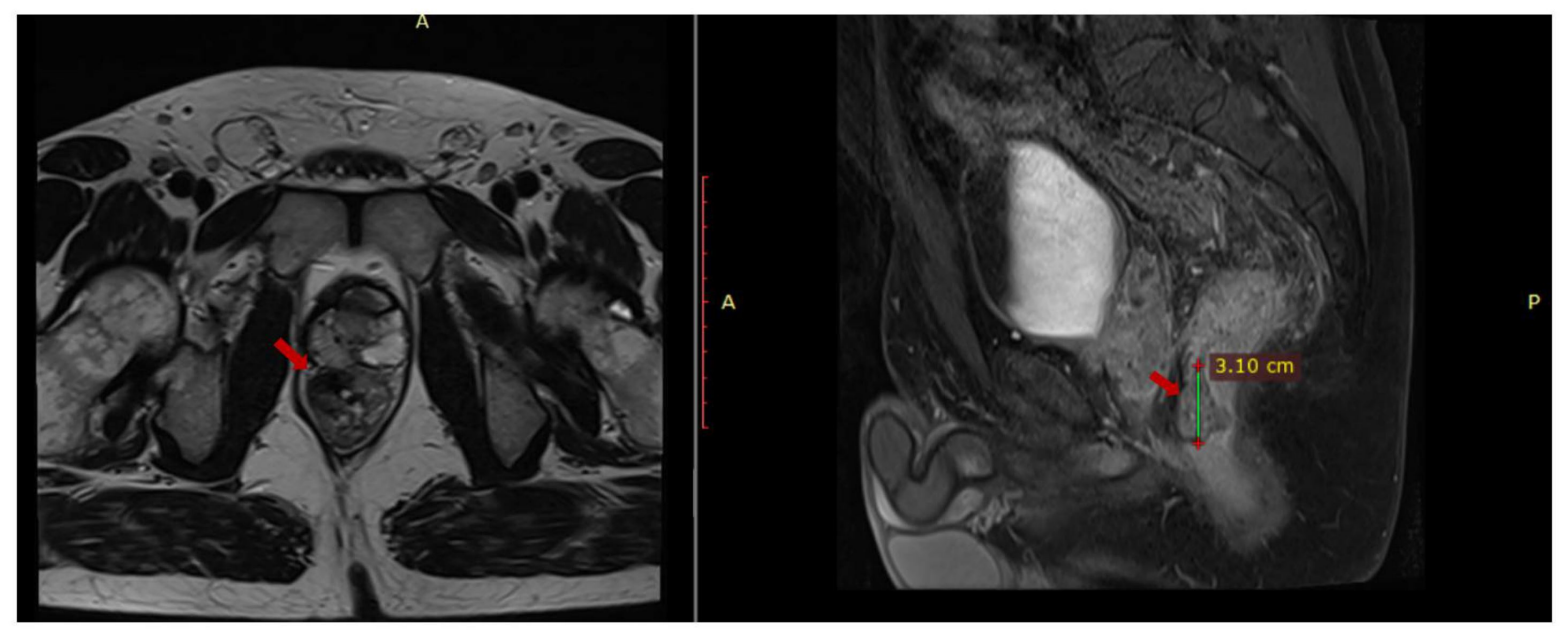
Pelvic MRI axial T1 and sagittal T2 sequences depict contrast-enhanced fibrous tissue in the rectum that adheres to the prostate.

An abdominal and pelvic MRI was performed in March 2023 and it identified circumferential parietal thickening of the rectal wall (1.2 cm), a fibrous lesion with microcalcifications (2.8 × 1.47 × 3.08 cm) adherent to the prostate, multiple mesorectal lymphadenopathies (up to 1.25 cm) and (1.97 × 0.87 × 1.7 cm) thick fluid collection located in contact with the anterior portion of the prostate (Figure 1).

On 14 March 2023, an endoscopic biopsy from the rectal lesion identified suspicious malignant signet ring cells, prompting an urgent trans-anal rebiopsy on 30 March 2023. In the distal rectum, at 6 o’clock, a tumor was identified with slight bleeding upon contact, extending to the lower pole of the prostate. A urological consultation was sought due to the involvement of the prostate. However, the urologist advised against performing a prostate biopsy, suggesting a high risk of hematoma, a concern that could complicate the patient’s clinical course, particularly given the patient’s intention to preserve fertility before initiating any treatment.

The chest CT (on 25 April 2023) did not show any pulmonary metastases.

### 2.3. Histology

Histological analysis revealed poorly differentiated endothelial-like cells arranged in irregular vascular channels. These cells exhibited atypical, pleomorphic nuclei, prominent mitoses, and spindly morphology.

Immunohistochemical testing (April 2023) showed positive staining for ERG, a marker associated with endothelial cells, and strong expression of CK AE1/AE3, indicating epithelial-like differentiation. The lymphoid and neural origins were excluded by the lack of CD20, CD3 and SOX10 expression, thus confirming the endothelial origin. Furthermore, aligning with the tumor’s aggressive behavior, the Ki-67 index of 80% underlines its high proliferative rate.

### 2.4. Differential Diagnosis and Pathological Discussion

The diagnosis of rectal angiosarcoma posed a significant diagnostic challenge given its rare occurrence and histopathological overlap with other malignancies [13].

#### 2.4.1. Soft Tissue Sarcoma

The primary differential diagnosis includes other soft tissue sarcomas, such as malignant fibrous histiocytoma, leiomyosarcoma, and undifferentiated pleomorphic sarcoma, all of which can exhibit pleomorphic, spindled, and mitotically active cells. However, the presence of rudimentary vascular channels, endothelial cell morphology, and the immunohistochemical profile, including ERG positivity, helps to distinguish angiosarcoma from these other sarcomas [13,14].

#### 2.4.2. GIST

Gastrointestinal stromal tumors (GISTs), which may also display spindle cell morphology, can be differentiated through the absence of endothelial markers and the positive staining for CD117 (c-KIT), which is typically absent in angiosarcoma [14].

#### 2.4.3. Malignant Melanoma

Malignant melanoma should also be considered in the differential diagnosis, particularly given the possibility of a rectal mass presenting with bleeding. However, melanoma typically expresses S100 and HMB-45, markers not present in angiosarcomas [14].

#### 2.4.4. Lymphoma

Lymphoma, particularly extranodal lymphoma, may imitate the aspect of angiosarcoma due to its mitotically active, pleomorphic cells; nevertheless, the CD20 and CD3 markers are typically positive, a feature absent in angiosarcoma [13].

#### 2.4.5. Other Vascular Tumors

Furthermore, other vascular tumors, such as hemangioendothelioma or Kaposi’s sarcoma, may share similar characteristics but they are less aggressive, have a clear clinical presentation, and exhibit a different immunophenotype [13].

The rarity of colorectal angiosarcoma poses significant diagnostic and therapeutic difficulties. Due to the lack of comprehensive clinical data, management protocols remain undefined, and treatment is often individualized. A detailed histological and immunohistochemical analysis are critical for confirming the diagnosis, as imaging alone cannot reliably distinguish angiosarcoma from other rectal malignancies [13,14] (Table 1).

### 2.5. Staging and Final Diagnosis

The NCCN guidelines classify angiosarcoma as a malignant vascular tumor and place it in the soft tissue sarcoma category [15]. Therefore, for this case, the American Joint Committee on Cancer (AJCC) Staging System for Soft Tissue Sarcoma of the Retroperitoneum (8th ed., 2017) was used [16,17] for the TNM classification, resulting in stage III (cT2 cN1 cM0).

### 2.6. Treatment

Following a comprehensive multidisciplinary team (MDT) discussion, a treatment plan was formulated.

CT simulation was performed in supine position with ≤3 cm slice thickness, fused with diagnostic MRI for accurate target delineation. Bladder filling techniques were employed to minimize bowel movement, and CBCT (cone-beam CT) was used daily for alignment verification.

The gross tumor volume (GTV) was created taking into account MRI contrast-enhanced fibrous tissue in the rectum and the extension to the prostate.

The clinical target volume (CTV) was calculated using the Valentini et al. guideline of target delineation, which included the rectum, mesorectum, internal iliac lymph nodes, presacral lymph nodes, obturator lymph nodes and the prostatic extension, with a CTV to PTV (planning target volume) of 0.5 cm. The external iliac nodes were excluded even though the adherence to the prostate was described, focusing more on tumor origin and histopathologic subtype [18,19,20,21].

**Figure 2:**
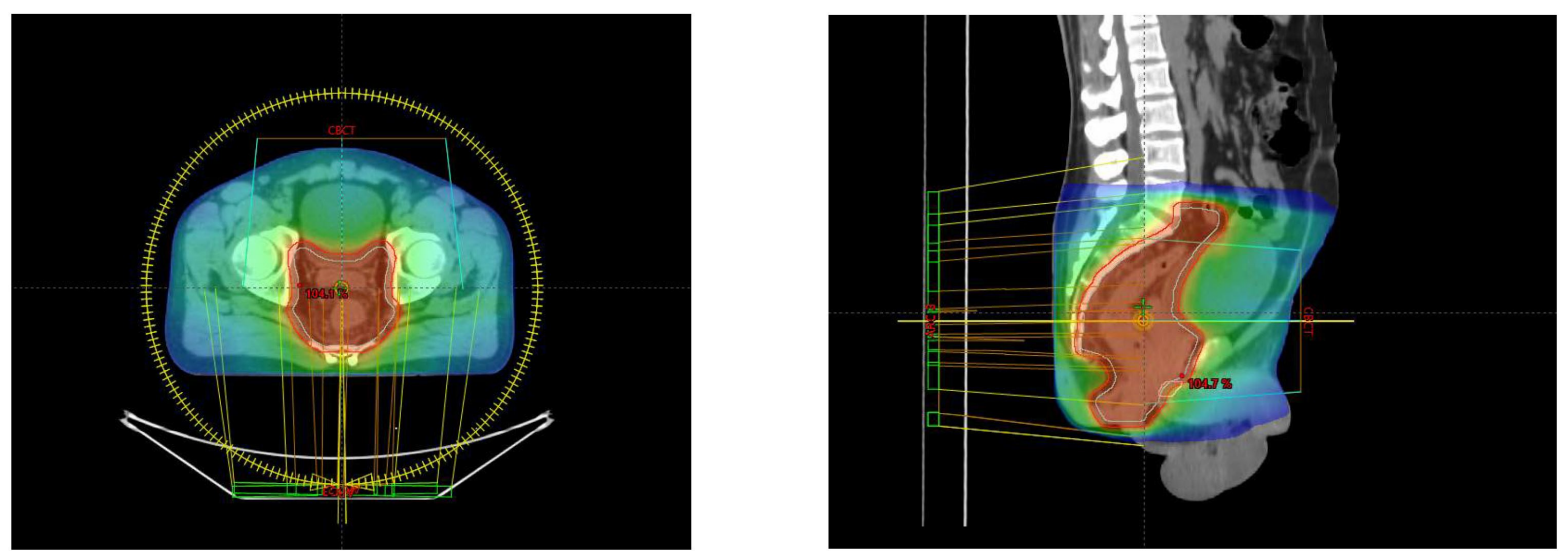
Axial and sagittal perspective of the treatment planning.

From 10 May to 21 June 2023, the patient underwent VMAT radiation therapy, receiving a total dose of 54 Gy in 30 fractions, 1.8 Gy/fraction, daily, with a pause on Saturday–Sunday (Figure 2).

The case was discussed in the MDT and surgical intervention was recommended. The patient underwent laparoscopic-assisted abdomino-perineal rectal amputation with a left-sided terminal colostomy.

Postoperative histopathologic examination revealed reactive changes induced by neoadjuvant therapy with relatively rare atypical-labeled cells present at the tumor bed. No positive pathologic lymph nodes were identified. No atypical cells were identified at proximal and circumferential resection margins (yp T0 yp N0 cM0).

### 2.7. Follow-Up

After the surgical intervention, the patient underwent two follow-up MRI exams to monitor the progress of the disease and assess for potential recurrence or metastases.

After more than 1 year of follow-up with serial MRI scans, the most recent imaging conducted in October 2024 at 6-month intervals revealed no sign of disease recurrence or metastatic progression. The pelvic cavity remains free of any signs of local recurrence. Other abdominal and pelvic organs, including the liver, pancreas, kidneys and spleen, continue to appear normal, and no new metastatic or secondary lesions have been identified. The patient’s status remains stable, with no new concerning findings on the MRI.

## 3. Discussion

### 3.1. Surgeon’s Perspective

From a surgical standpoint, this case presented distinctive challenges taking into account the tumor’s highly vascular nature and proximity to critical pelvic structures such as pelvic autonomic nerves, urogenital organs and vascular plexuses.

In order to achieve oncological safety while minimizing morbidity, the surgical approach was to perform a laparoscopic rectal amputation with colostomy. The overall objective and main challenge was to obtain a negative resection margin (R0), which is why the surgical team opted for laparoscopic-assisted abdomino-perineal rectum amputation with left terminal colostomy.

In order to allow simultaneous access to the abdominal and perineal region, the patient was positioned in a modified lithotomy position. Following the established pneumoperitoneum, a standard laparoscopic approach was used with the placement of five trocars. After the initial exploration confirmed no peritoneal spread, dissection began with a medial-to-lateral mobilization of the left colon, meticulously identifying and preserving the left ureter and gonadal vessels. For optimal hemostasis, the inferior mesenteric artery was ligated. A sufficient colonic length for a tension-free colostomy was ensured, followed by sharp dissection of the rectum. The procedure required careful navigation of fibrotic tissues so as to protect adjacent structures, particularly the pelvic autonomic nerves. Once the rectum was mobilized down to the level of the pelvic floor, the procedure transitioned to the perineal phase with the en bloc excision of the anorectal complex without breaching oncologic margins. An end colostomy was carried out in the left lower quadrant. The perineal wound was closed by primary intention. Intraoperative assessment did not reveal any macroscopic invasion of the prostate and a biopsy was avoided due to concerns about hematoma risk and the patient’s wish to preserve fertility.

Anatomical and technical considerations included pelvic autonomic nerve-sparing dissection due to the proximity of these structures, preservation of ureteral integrity, optimal hemostasis and early vascular control, with regard to oncological safety. Compared to a routine rectal amputation, this case called for a wider and more aggressive approach to achieve negative margins. Given the vascular behavior of the tumor, the surgical attitude prompted an earlier arterial ligation. Plastic surgeons were kept on standby if the perineal defect needed a flap reconstruction, though primary closure was accomplished.

### 3.2. Medical Physicist’s Perspective

From a dosimetric perspective, due to the large extent of the volume in the cranio-caudal direction and the multileaf collimator (MLC) limitation of the linear accelerator (LINAC), the chosen optimal number of full treatment arcs was 3. This treatment was delivered on a TrueBeam STx, with the photon beam of 6X (6MV).

Due to the presence of the organs at risk (OARs), specifically the “Testis” and “Penile Bulb”, a tighter collimation was preferred in this region to obtain doses as small as possible, keeping in mind the patient’s age. Regarding the dose constraints for the “Bladder”, “Bowel” and “Femoral Heads”, there were no difficulties in achieving the desired targets, especially with bladder filling and the bowel being pushed away from the PTV.

For the PTV coverage, the following constraints were used: V95% > 98% (obtained 99.4%) and V105% < 2% (obtained 0.05%), with a hotspot of 107.4%.

For OARs, the following dose constraints were used:

• Bladder—V40 Gy < 50% (obtained 29.07%); Dmax < 105% (obtained 104.4%).
• Femoral Heads (each)—Dmax < 45 Gy (obtained left 39.3 Gy; right 38.5 Gy).
• Bowel—V45 Gy < 195 cm^3^ (obtained 33.9 cm^3^); Dmax < 105% (obtained 104.5%).
• Penile Bulb—D50% < 50 Gy (obtained 52.2 Gy).
• Testis—D50% < 3 Gy (obtained left 1.47 Gy; right 1.7 Gy).

This highlights the technical challenges of balancing optimal tumor control with the preservation of organ function in young patients undergoing pelvic irradiation for rare sarcomas.

### 3.3. Oncologist’s Perspective

No evidence-based treatment recommendations can be provided for specific angiosarcoma subtypes and patients with this rare condition should be referred to specialized centers. For patients with localized disease, radical surgery with complete (R0) resection is the primary treatment of choice. Wide margins are advised due to the invasive and frequently multifocal nature of angiosarcomas.

There is a lack of prospective data on neoadjuvant/adjuvant chemotherapy for patients with angiosarcomas. A retrospective analysis including 362 patients with primary angiosarcomas of any site showed that neoadjuvant/adjuvant chemotherapy may improve outcomes for patients with larger tumors (>5 cm) and/or higher risk of death [22]. Anthracyclines, ifosfamide and more recently taxanes and gemcitabine are all active chemotherapy agents in angiosarcomas.

Adjuvant chemotherapy was not considered for the patient presented, given the good clinical and pathological response obtained with neoadjuvant radiotherapy (complete pathological response) and also taking into consideration the patient’s desire to preserve fertility.

### 3.4. Contribution to Medical Knowledge

The body of medical literature comprises around 40 documented cases of colorectal angiosarcoma, on account of the scarcity of primary angiosarcoma of the gastrointestinal tract.

In order to provide context and comparison, two prior published cases of gastrointestinal angiosarcoma were reviewed and analyzed alongside the case elaborated above to highlight similarities and differences in clinical presentation, treatment approach and outcomes. In the first case reviewed, one patient with sigmoid angiosarcoma [9] showed rapid disease progression that resulted in liver metastases shortly after surgery and resulted in death during the first 6 months, demonstrating the aggressive behavior according to this example and outlining the median lifespan of 7 months as stated previously. The second case reviewed [23] showed a positive surgical outcome for a patient with caecal angiosarcoma, which provided results comparable to the patient reported herein who remains disease-free beyond one year after treatment. The symptoms of rectal bleeding, urgency and constipation of the patient described herein presented in a non-specific manner of presentation, which resulted in delayed diagnosis.

Furthermore, the case reported in this article lacked any known radiation exposure history, which is known to increase angiosarcoma development risk, impacting the tumor’s idiopathic nature. Moreover, the case presents an interesting clinical scenario because it involved a 41-year-old male patient who does not fit the typical demographic profile of colorectal angiosarcomas, which usually affect women in their mid-50s. Additionally, the histological diagnosis of angiosarcomas proves challenging when using small biopsy samples because these tumors resemble poorly differentiated carcinoma of gastrointestinal stromal tumors (GISTs), proving that immunohistochemical testing is essential for obtaining an accurate medical diagnosis.

In addition to contributing to the limited body of literature on colorectal angiosarcomas, this report sheds further light on the aggressive behavior and diagnostic complexity of rectal angiosarcomas, the critical role of achieving complete (R0) surgical resection, ideally supported by a multidisciplinary approach that includes surgery, radiotherapy and ongoing close imaging surveillance, also offering new insights into managing such tumors in a younger, non-irradiated patient with the intention of preserving fertility, which is rarely discussed in the few existing case series.

Future case reports together with multicenter collaborations will be crucial to enhance the understanding of prognostic indicators and optimize treatment strategies, and eventually guide evidence-based management for patients facing rare malignancies.

## 4. Conclusions

Rectal angiosarcoma accounts for one of the rarest malignancies and owing to its aggressive behavior, it requires prompt diagnosis and multidisciplinary approach for optimal outcomes. This case illustrates the importance of collaboration amongst surgeons, oncologists, radiation oncologists, radiologists and pathologists in managing such complex conditions.

## Figures and Tables

**Table 1:** Differential diagnosis.

Condition	Key Features for Exclusion
Soft Tissue Sarcoma	Negative for ERG markers and lack of vascular channels.
Gastrointestinal Stromal Tumor	Lack of CD117 (c-KIT) expression.
Malignant Melanoma	Absence of S100 and HMB-45 markers.
Lymphoma	Negative CD20 and CD3, excluding lymphoid origin.
Other Vascular Tumors	Less aggressive presentation and differing immunophenotype (e.g., hemangioendothelioma).

## Data Availability

Data supporting reported results can be requested from the corresponding author due to privacy concern.

## Abbreviations

The following abbreviations are used in this manuscript:

ERG	ETS-Related Gene
GIST	Gastrointestinal Stromal Tumor
IMRT	Intensity-Modulated Radiation Therapy
VMAT	Volumetric-Modulated Arc Therapy
CTV	Clinical Target Volume
PTV	Planning Target Volume
CBCT	Cone-Beam CT
MDT	Multidisciplinary Team
MLC	Multileaf Collimator
LINAC	Linear Accelerator
OARs	Organs at Risk
